# Acupuncture combined with tamsulosin hydrochloride sustained-release capsule in the treatment of chronic prostatitis/chronic pelvic pain syndrome: A study protocol for a randomized controlled trial

**DOI:** 10.1097/MD.0000000000019540

**Published:** 2020-03-20

**Authors:** Ji-Sheng Wang, Jie Yang, Sheng Deng, Xu-Dong Yu, Bing-hao Bao, Rui-Jia Liu, Hai-Song Li

**Affiliations:** a Graduate School of Beijing University of Chinese Medicine; b Department of Andrology, Dongzhimen Hospital, Beijing University of Chinese, Beijing, China; c Beijing Fengtai Maternal and Child Health Hospital,Being, China; d The Fist Department of Neurology, Dongzhimen Hospital, Beijing University of Chinese Medicine, Beijng, China.

**Keywords:** acupuncture, chronic pelvic pain syndrome, chronic prostatitis, protocol, randomized controlled trial

## Abstract

**Background:**

Chronic prostatitis/chronic pelvic pain syndrome (CP/CPPS) is a common urinary system disease in men. As part of traditional Traditional Chinese medicine, acupuncture has been widely used in clinical practice. In order to evaluate the exact effect of acupuncture on the clinical efficacy of CP/CPPS, this experiment uses randomized controlled experiments.

**Methods/design:**

This pragmatic randomized controlled trial will recruit 166 patients who are diagnosed with CP/CPPS. Simple randomization to conventional drug treatment with a 1:1 allocation ratio will be used. Ten 30-minute acupuncture sessions will be provided to patients assigned to the Intervention group. All participants will continue to receive conventional drug treatment. The selection of outcomes will be evaluated by Health's Symptom Score Index (NIH-CPSI) score at week 4.

**Discussion:**

This trial may provide evidence regarding the clinical effectiveness, safety, and cost-effectiveness of acupuncture for patients with CP/CPPS.

**Trial registration:**

ClinicalTrials.gov, ChiCTR1900021132, Registered on 29 January 2019

## 1 Introduction

Chronic prostatitis/chronic pelvic pain syndrome (CP/CPPS) is a common urinary system disease in men.^[1,2]^ The main symptoms of CP/CPPS include recurrent, long-term pelvic floor area, lumbosacral pain and discomfort, and varying degrees of lower urinary tract symptoms (frequency, urgency, dysuria, endless urination, etc.). Some patients also experience dizziness, fatigue, memory loss, sexual dysfunction, and even depression.^[3,4]^ Epidemiological surveys show that 2.2% to 13.8% of adult men worldwide are affected by this, while 30% to 50% of men are affected at some time in their lives.^[5]^ Related literature reports that the prevalence of CP/CPPS and related symptoms in China is as high as 46.6%. CP/CPPS not only causes great pain to the patient's body and mind, but also causes severe social burden due to its multiple and repetitive nature.^[6]^ In view of this, the National Institutes of Health (NIH) has currently listed CP with myocardial infarction, unstable angina pectoris, and active Crohn disease as the most serious chronic diseases that affect residents’ quality of life.^[7,8]^

As part of traditional Traditional Chinese medicine (TCM), acupuncture has been widely used in clinical practice. Recent studies have shown that acupuncture is effective in reducing chronic pain and anti-fibrosis in the pelvic floor area. Studies have shown that acupuncture stimulates the patient's analgesic system by stimulating related acupoints, accelerates the production of endogenous opioid peptides in the central nervous system and acts on the corresponding receptors, thereby achieving the effect of peripheral analgesia.^[9,10]^ Anti-inflammatory effects can also be achieved by increasing β-Ep levels in inflammatory tissues and serum.^[11,12]^ TCM believes that acupuncture can adjust the balance of Qi and blood in the human body, and stimulates acupuncture points to improve human function. It is more and more popular with doctors and patients for its unique advantages of simplicity, convenience and effectiveness.

In order to evaluate the exact effect of acupuncture on the clinical efficacy of CP/CPPS, this experiment uses randomized controlled experiments. We will verify the clinical efficacy of acupuncture treatment for CP/CPPS by observing the effects of acupuncture combined with tamsulosin hydrochloride sustained-release capsules on the improvement of symptoms and mental health in patients with CP/CPPS.

## 2 Methods/design

### 2.1 Study design and settings

**Figure 1:**
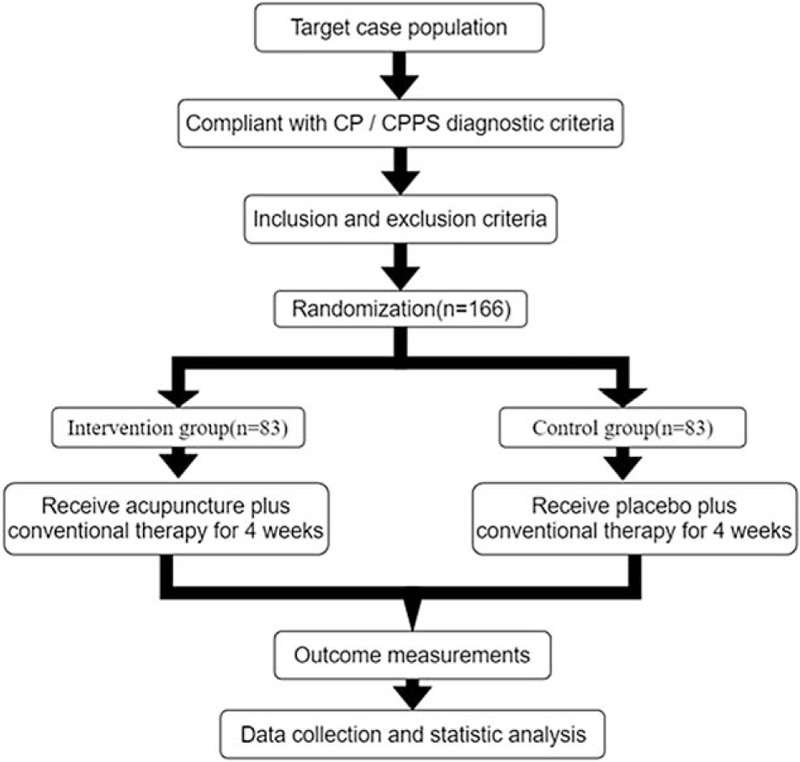
Study design flow chart.

A brief flowchart of the entire study is shown in Figure 1. We will perform a 2-group, randomized, single-blind, placebo-controlled, multi-center trial that will evaluate the efficacy and safety of acupuncture for patients with CP/CPPS. This study will use a completely random grouping and parallel control observation design method. We will ensure the balance of the baseline data of the two groups through a sufficient sample size and a completely randomized grouping method. This study will be approved by the Ethics Committee of Dongzhimen Hospital Affiliated to Beijing University of Chinese Medicine. We will not begin recruiting at other centers in the trial until local ethical approval has been obtained. This study is registered at http://www.chictr.org.cn/showproj.aspx?proj=35685 (ChiCTR1900021132).The protocol includes elements recommended in the Standard Protocol Items: Recommendations for Interventional Trials checklist (Additional file 1).

### 2.2 Setting and participants

This study will be conducted in China. Patients will be recruited from Urology/Andrology departments of Dongzhimen Hospital. We will enroll participants based on the following inclusion criteria:

1. Those who meet the CP / CPPS western medical diagnostic criteria;
2. Those whose disease course exceeds 3 months;
3. Twenty years old ≤ age ≤ 50 years old;
4. Patients who have been treated before the consultation can be included in the study after at least 2 weeks of drug withdrawal;
5. Those who have signed informed consent.

Participants who meet the following exclusion criteria will not be enrolled:

1. Patient with a history of pelvic surgery;
2. Persons with abnormal development or infection of urogenital system;
3. Prostate tuberculosis, benign prostatic hyperplasia and prostate cancer, acute urethral syndrome;
4. Patients with severe physical illness, systemic failure, and allergies to tamsulosin hydrochloride sustained-release capsule drugs;

### 2.3 Randomization, allocation concealment, and blinding

The web-based online randomization system to be used in this trial was provided by an independent academic data management center at Dongzhimen Hospital Affiliated to Beijing University of Chinese Medicine. The attending Physician will identify eligible patients according to the inclusion and exclusion criteria. Informed consent will be taken by the attending physician, and participants will be referred to a research coordinator who will randomly assign them to the intervention or control arm. The randomization list is kept by the biostatistician and research coordinator until the end of the study to ensure allocation concealment; therefore, the data analysts will be kept blinded to the allocation. The participants will be instructed not to disclose the allocation to the attending physician.

### 2.4 Interventions

Intervention group: acupuncture combined with tamsulosin hydrochloride sustained-release capsules. The acupuncture points will be selected as *Shenshu*, *Zhongliao,* and *Sanyinjiao*. The patient will be placed in the supine position. After routine disinfection of the local skin, a straight puncture of 25 to 40 mm will be performed, and twisting and replenishing and reducing methods were performed. The needle will be left for 30 minutes after getting gas, 3 times a week. The course of treatment was 4 weeks.

Control group: Tamsulosin hydrochloride sustained-release capsules (Brand name: Harlot, manufactured by Astellas Pharmaceutical Co., Ltd., specifications: 0.2 mg × 10 capsules), 0.2 mg each time, orally before bedtime, once a night. The course of treatment was 4 weeks.

Treatment will be performed once a week for a total of 4 weeks at approximately the same time in the afternoon. No restrictions will be imposed on the standard treatments for pain or any other disease during this study. If the patient is discharged according to the clinical evolution or is deceased, he/she will be excluded from this study. Further, occurrence of a serious adverse event or withdrawal of consent will result to study exclusion. During the treatment, all patients should not take other drugs that have a therapeutic effect on the disease.

### 2.5 Data collection

The study data collection process is outlined in Table 1.

**Table 1:**
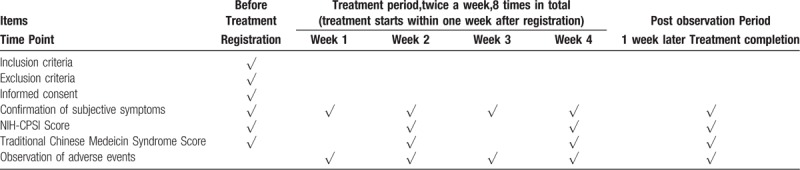
Treatment schedule and outcome measures.

### 2.6 Primary outcomes

The National Institutes of Health's Symptom Score Index (NIH-CPSI) score for CP/CPPS will be used as the primary outcome measure. Every dimensions (degree of pain, urinary symptoms, quality of life) of NIH-CPSI will be evaluated, and the lower the total score, the lighter the clinical symptoms of the patient.

### 2.7 Secondary outcomes

The second outcome measure is based on TCM syndrome evaluation criteria.(1) Healing: The clinical symptoms and signs of TCM disappear or almost disappear, and the syndrome score is reduced by ≥90%; (2) Significant effect: The clinical symptoms and signs of TCM are obviously improved, and the syndrome score is reduced by ≥60%; (3) Effective: Chinese medicine Clinical symptoms and signs have improved, syndrome scores decreased by < 60%, but ≥30%; (4) Invalid: The clinical symptoms and signs of TCM were not improved, even worse, and the syndrome score was reduced by < 30%. Integral variation formula (Nimodipine method: [(pre-treatment score - post-treatment score) ÷ pre-treatment score] × 100%.

### 2.8 Statistical consideration

#### 2.8.1 Sample size

Based on the results of our previous study, the effective rate of CP/CPPS in the intervention group was P1 = 0.80. The PSD effective rate of the control group was P2 = 0.65 [(two-sided type I error rate of 0.05, α = 0.05); (one-sided type I error rate of 0.10, β = 0.10)]. Substituting f (α, β) into the formula is as follows: n1 = n2 = 10.5 × (0.8 × 0.2 + 0.65 × 0.35) ÷ 0.152 = 75. The lost follow-up rate of patients will be controlled at 10%, so an additional 8 cases are added to each group. n1 = n2 = 83, that is, 83 cases will be taken from each of the intervention group and the control group. According to Cohen, this effect size is considered “moderate”.

#### 2.8.2 Analysis set

The Full Analysis Set will be used for all primary analysis. Per Protocol Set analysis will be performed for evaluating the sensitivity of results.

#### 2.8.3 Statistical analysis

Data management uses EXCEL software to build a database, double entry, check for outstanding values, and lock. Statistical analysis will be performed using SPSS 25.0 software for statistical analysis. The normality of the measurement data is tested. The data obeying the normal distribution is Student's t test, which is expressed by mean ± standard deviation. The data not obeying the normal distribution is rank sum test. And marginal homogeneity test; count data are expressed by rate and composition ratio, and comparison is performed by chi-square test; repeated measurement data are expressed by mean ± standard deviation, intra-group comparison is performed by analysis of variance of repeated measurement data, and inter-group comparison is by multivariate analysis of variance (MANOVA). *P* ≤ .05 indicates that the difference is statistically significant.

### 2.9 Quality control and trial management

The management structure will comprise the principal investigator (PI), a trial management group, and a data monitoring committee. The trial management group will be responsible for conducting the trial and will meet monthly to discuss the trial progress. The PI will visit each collaborative hospital for face-to-face meetings and to share information to promote patient recruitment. The data monitoring committee will review safety and efficacy data. All data will be monitored every month through a central monitoring method. Additional monitoring may be performed at the discretion of the monitoring manager. The data monitoring committee have met once prior to the start of patient recruitment. At least twice per year, participating investigators, research assistants, and research nurses will be required to attend a training workshop on clinical research to ensure strict adherence to the study protocol and familiarity with the trial administration process. The data collected in this trial will comprise information recorded in case report forms and questionnaires. Data quality will be checked regularly by research assistants and overseen by monitors; all modifications will be marked on case report forms and data managers will recheck the data before they are officially logged. The database will be locked after all data have been cleaned. If participants withdraw from the trial during the study period, the reasons will be documented and the dropout rate will be statistically analyzed.

### 2.10 Feasibility analysis

The hospital has formulated related systems, such as scientific research organization management, to provide guarantee measures for the organization and management of the subject. In strict accordance with the relevant requirements of the superiors, the financial department is responsible for management and supervision, and establishes accounts according to items, which are used exclusively by the project team. Carry out research as planned, actively accept the guidance and supervision of relevant higher-level management departments, and submit the Annual Summary Report on the Implementation of Scientific Research Projects to management departments every year. The Scientific Research Department is responsible for the daily management during the implementation of the project, helping to solve practical difficulties, inspecting and supervising the research progress, and establishing subject files; major personnel changes and major changes in research plans and programs must be reported to the superior management department Approval. In order to ensure that the team leader and members have enough time and energy to devote themselves to the research, the college has formulated related systems and strictly implemented the responsibility system for the team leader.

## 3 Discussion

The etiology of CP/CPPS is complex, and its pathogenesis has not been fully elucidated so far. There are reports in the literature that there is some connection between prostate hyperplasia and CP / CPPS. Modern medicine does not have a clear treatment plan for CP/CPPS.^[13]^ At present, antibiotics and alpha blockers are often used for treatment. In recent years, clinicians have gradually realized the heterogeneity of chronic prostatitis, that is, different people have different etiology, different clinical manifestations, different disease processes and different responses to treatment.^[14,15]^ In addition, due to the special nature of the prostate anatomy—the inner membrane is deeply wrapped, it is easy to cause local microcirculation disturbance, drainage is blocked, the drug is difficult to reach the lesion, and it is unable to fully achieve the effect.^[16]^ important reason. Therefore, in the treatment of chronic prostatitis, while emphasizing comprehensive treatment, more and more attention is paid to individualized treatment, and treatment options are selected based on individual specific clinical manifestations. Chronic prostatitis / chronic pelvic pain syndrome (CP/CPPS) accounts for 90% to 95% of prostatitis.^[17,18]^ Although there are many treatments for CP / CPPS, there are no specific treatments, and the treatment results are not satisfactory.^[19]^ Lack of a unified and standardized treatment plan.

At present, oral medication for chronic prostatitis is the most important treatment method, and the effect is significant. However, there are some intractable problems with oral medication, such as higher treatment costs, longer treatment periods, and long-term gastrointestinal stimulation in patients. This leads to many patients not being able to adhere to treatment, which ultimately affects the clinical efficacy, and even leads to the loss of previous achievements. It is therefore important to explore new treatment options. Therefore, we will evaluate the clinical effectiveness and safety of acupuncture in the treatment of CP/CPPS. We hope that the results of this study will provide clinicians with the basis for acupuncture treatment of CP/CPPS and more treatment options.

## 4 Trial status

At the time of submission of the manuscript, the study had not yet begun to recruit subjects.

## Acknowledgments

The authors would like to thank all the trial participants. The authors are grateful for the support for this study: trial coordinating team, surgical staff, nurses, and research departments.

## Author contributions

JSW, JY, XDY, SD, and RJL designed the study protocol and drafted the manuscript. HSL reviewed the study protocol and drafted the manuscript. BHB is responsible for the statistical design and analysis as trial statistician. All authors carefully read and approved the final version of the manuscript.
